# Non-surgical interventional treatments for knee osteoarthritis: The need for different perspectives, cross-specialty collaboration, and preclinical and translational research

**DOI:** 10.1016/j.inpm.2024.100385

**Published:** 2024-01-15

**Authors:** Steven P. Cohen, Ludger Gerdesmeyer, Zachary L. McCormick

**Affiliations:** Departments of Anesthesiology, Neurology, Physical Medicine & Rehabilitation, Psychiatry and Behavioral Sciences, and Neurosurgery, Northwestern University Feinberg School of Medicine, Chicago, IL, USA; Departments of Anesthesiology and Physical Medicine & Rehabilitation, Walter Reed National Military Medical Center, Uniformed Services University of the Health Sciences, Bethesda, MD, USA; Department of Orthopaedic Surgery & Traumatology, Public Health Clinic, Kiel, Germany; Division of Physical Medicine & Rehabilitation, University of Utah School of Medicine, Salt Lake City, UT, USA

In his eloquent commentary on developing a new paradigm for conceptualizing and treating pain due to knee osteoarthritis (KOA), Dr. Devor elucidates several important points often overlooked by interventionalists [1]. Yet, by the same token neuroscientists, who do not treat patients, may not have the foundational knowledge germane to performing effective interventional pain procedures. The goal, as set forth in the article by Devor, is to enhance communication between these often-isolated groups in order to come up with new ways to think about and treat chronic pain. As a multidisciplinary group of academic physicians, we appreciate and encourage this dialogue.

At the beginning of his commentary, Dr. Devor emphasizes that “pain is not due to bones, cartilage, tendons or periosteum. Pain is due to nerves … tissue innervation and, more to the point, the trains of electrical impulses that carry the resulting pain signal from peripheral tissues into the central nervous system (CNS).” However, this is fundamentally at odds with the nearly universally-accepted framework for the classification of pain. Nociceptive pain, for which osteoarthritis is a prototype, is defined by the International Association for the Study of Pain (IASP) as “activity in neural pathways secondary to actual or potentially tissue-damaging stimuli.” In contrast, neuropathic pain is defined as “pain caused by damage or disease affecting the somatosensory nervous system.” [2] The distinction is critical because, as Dr. Devor himself has acknowledged, understanding mechanisms forms the groundwork for rational, evidence-based pain treatment [3]. Most pain due to KOA falls into the category of nociceptive, though a substantial percentage of patients have a nociplastic component and are thus at high risk to fail any interventional treatment [4]. A smaller proportion of patients also have concrete evidence of nerve damage (neuropathic pain).

In chronic OA, biomechanical stress results in repetitive microtrauma to the collagen-bone skeletal system. Mechanically-derived input on osseous and collagen structures is defined as mechano-transduction, which can induce adaptation of the intrinsic bone innervation. Intrinsic bone innervation refers to the presence of nerves within bone itself. In addition to bones, dynamic tissues such as muscles, cartilage, ligaments, and tendons also provide structural support to joints; with the exception of cartilage which is devoid of blood vessels, lymphatics and nerves, these elements are intertwined in a complex, interactive network containing blood vessels, nerves, and a host of immune and other related systems. Nerves in bone tissue play crucial roles in various physiological processes, including pain perception, bone remodeling, and the regulation of blood flow. Changes in mechano-transduced impulses can result in adaptation of neuroceptive bone structures, including maladaptive changes affecting extrinsic and intrinsic neurons. These can include amplification of pain signals, disinhibition of modulatory processes, and abnormal reinnervation patterns involving C-fibers sprouting into territory affected by microtrauma leading to expansion of receptive fields and mechanical allodynia [5].

As a renowned neuroscientist, Dr. Devor makes astute observations in his commentary regarding the intrinsic and extrinsic innervation of the knee joint, using analogies easily understood by surgeons and pain physicians (e.g., basivertebral nerves as intrinsic innervation, the medial branches of dorsal rami as extrinsic innervation, the tooth decay and root canal analogy). Yet, the distinction between intrinsic and extrinsic innervation is not black and white or binary, like pregnancy. Some experts have described the different categories of pain as points on a continuum, as joint, subchondral cancellous bone, and soft tissue injury generally involves damage to microscopic nerve endings, and most types of neuropathic pain also involve tissue injury (e.g., ischemia and ulcers complicating diabetic neuropathy, cutaneous inflammation and tissue damage in acute herpes zoster and postherpetic neuralgia) [6]. This paradigm is supported by studies demonstrating efficacy for anti-inflammatory drugs for neuropathic pain and others suggesting the benefit of membrane stabilizers for nociceptive pain [[7], [8], [9]].

Dr. Devor adroitly highlights the limitations of genicular nerve radiofrequency ablation (RFA), which stem not only from not targeting enough of the “extrinsic” nerve supply in many of the early studies [[10], [11], [12]], but also from possible failure to interrupt the intrinsic innervation. However, it is axiomatic that all intrinsic innervation stems from extrinsic innervation, which is easier to identify and target. For example, anatomical dissection studies now demonstrate multiple medial branches at cervical spine levels, which not only contain ascending and descending branches but also countless small filaments that branch out from these extrinsic nerves and form the intrinsic nerve supply to the facet joints [13]. By capturing the extrinsic afferent nerves using radiofrequency ablation, cervical facet joint pain can be abolished [14]. Whereas the knee joint contains a more complex extrinsic nerve supply relative to a cervical facet joint, this does not preclude the ability to greatly reduce pain symptoms when accounting for this complexity with updated, comprehensive approaches to genicular RFA [15]. Additional clinical outcome literature will be useful in confirming early findings from pilot work and non-prospective predictive modeling studies [[16], [17], [18], [19], [20]], and indeed, a large-scale multi-center prospective study is underway [21].

Although biologically plausible, injecting polymethyl methacrylate (PMMA) into the epiphyseal bone end is almost certain to have unintended consequences. Leakage of cement is extremely common for other pain-relieving procedures such as vertebral augmentation [22]. Whereas a small amount of leakage is generally inconsequential, the implications of leakage into a mobile, synovial joint space and its effects on future surgery (e.g., joint replacement) are unknown, and severe complications resulting from leakage into joints have already been reported [23]. Moreover, the long-term effects of violating intact bone with a large trocar are unclear, but existing literature suggests that the use of a large trocar within a vertebral body may increase the risk of subsequent fracture in patients with compromised bone density [24]. It is also notable that a very large volume of cement would likely need to be deposited into the femoral condyles in order to accomplish destruction of the relevant intrinsic nerves, particularly when compared to the volume of cement typically used during vertebral augmentation procedures. It is not challenging to imagine the negative implications of applying a large volume of cement within the femoral condyles on gait mechanics and effects along the kinetic chain, particularly if performed on one but not both knees.

As an alternative, Dr. Devor proposes consideration of injecting neurolytic agents or widespread cauterization around the epiphyseal bone end. Although interest in peri-articular chemoneurolysis (targeting the extrinsic afferent nerve supply to the knee joint) has recently grown [[25], [26], [27]], injection of a chemoneurolytic agent into the trabecular bone of the femoral condyles to target the intrinsic nerve supply is not likely to work. Bone is highly vascular and injection of a liquid agent is likely to be washed away before thorough neurolysis could realistically occur. With regard to RFA, the scientific basis for this therapy is that a controlled lesion that captures an identifiable neural target (i.e., by ultrasound or electrostimulation) is likely to provide benefit for nociceptive pain without significant off-target side effects. Widespread periarticular cauterization of the perforating nerves circumferentially along the medial, lateral, and anterior aspects of the femoral and tibial epiphyses would be extraordinarily time intensive and may lead to penetration of the joint capsule with subsequent risk of septic arthritis. Additionally, numerous RFA needle passes in close proximity to the femoral and tibial epiphyses would likely place the tendon footprints of muscles attachments to these areas at risk of structural failure due to significant fenestration. Finally, if these epiphyseal perforating nerves were cut or mechanically disrupted in another way, neuroma formation and/or deafferentation pain would be a concern.

Perhaps the main value in this commentary is that Dr. Devor is thinking outside of the box, outside of the usual paradigm by which we have been treating joint pain for over 40 years since the first descriptions of radiofrequency ablation in the 1970's [28]. Similar to many interventions, radiofrequency ablation for facet and knee joints did not undergo rigorous clinical studies in animals before being trialed in humans, so that many of the technical (e.g., using small electrodes that create small lesions) and selection (e.g., prognostic block paradigms) factors were not optimized. Yet, Dr. Devor proposes making the same mistake that was made with radiofrequency ablation as it pertains to the knee, namely performing clinical without preclinical studies to validate the approach and confirm safety. But the key message that arises from Dr. Devor's commentary is clear: the clinical impact of intrinsic bone innervation is underestimated and should be a research and clinical focus-in that order-for neuroscientists, pain physicians and surgeons. Although the solutions proposed may not hold all of the answers that we are looking for, as Dr. Devor's commentary implies, the search-including in preclinical and translational studies-must continue.

## Funding statement

This work was partly supported (partial effort for SPC) by the U.S. Dept. of Defense, Uniformed Services University, Department of Physical Medicine & Rehabilitation, Musculoskeletal Injury Rehabilitation Research for Operational Readiness (MIRROR) (HU00011920011). This organization played no role in the preparation of this manuscript.

## Declaration of competing interest

The authors declare no conflicts of interest for this manuscript.

## References

[1] Devor M. (2024). Pain in osteoarthritis: driven by intrinsic rather than extrinsic joint afferents and why this should impact treatment. Interventional Pain Medicine.

[2] Cohen S.P., Vase L., Hooten W.M. (2021). Chronic pain: an update on burden, best practices, and new advances. Lancet.

[3] Devor M. (2018). Rethinking the causes of pain in herpes zoster and postherpetic neuralgia: the ectopic pacemaker hypothesis. Pain Rep.

[4] Fitzcharles M.A., Cohen S.P., Clauw D.J., Littlejohn G., Usui C., Häuser W. (2022). Chronic primary musculoskeletal pain: a new concept of nonstructural regional pain. Pain Rep.

[5] Gangadharan V., Zheng H., Taberner F.J., Landry J., Nees T.A., Pistolic J. (2022). Neuropathic pain caused by miswiring and abnormal end organ targeting. Nature.

[6] Cohen S.P., Mao J. (2014). Neuropathic pain: mechanisms and their clinical implications. BMJ.

[7] Yoon M.H., Yaksh T.L. (1999). The effect of intrathecal gabapentin on pain behavior and hemodynamics on the formalin test in the rat. Anesth Analg.

[8] Clarke H., Bonin R.P., Orser B.A., Englesakis M., Wijeysundera D.N., Katz J. (2012). The prevention of chronic postsurgical pain using gabapentin and pregabalin: a combined systematic review and meta-analysis. Anesth Analg.

[9] Vo T., Rice A.S., Dworkin R.H. (2009). Non-steroidal anti-inflammatory drugs for neuropathic pain: how do we explain continued widespread use?. Pain.

[10] Cohen S.P., Mishra P., Wallace M., Sellers A., Veizi E., Hurley R.W. (2023). Emperor's nakedness exposed: unmasking fairytales for genicular nerve radiofrequency ablation in knee osteoarthritis. Reg Anesth Pain Med.

[11] Conger A., Gililland J., Anderson L., Pelt C.E., Peters C., McCormick Z.L. (2021 Jul 25). Genicular nerve radiofrequency ablation for the treatment of painful knee osteoarthritis: Current evidence and future directions. Pain Med.

[12] Fogarty A.E., Burnham T., Kuo K., Tate Q., Sperry B.P., Cheney C., Walega D.R., Kohan L., Cohen S.P., Cushman D.M., McCormick Z.L., Conger A. (2022 May 1). The effectiveness of fluoroscopically guided genicular nerve radiofrequency ablation for the treatment of chronic knee pain due to osteoarthritis: A systematic review. Am J Phys Med Rehabil.

[13] Büsken F., Lataster A., Herrler A. (2022). The innervation of the cervical facet joints- an anatomical and histological approach. Clin Anat.

[14] Lord S.M., Barnsley L., Wallis B.J., McDonald G.J., Bogduk N. (1996). Percutaneous radio-frequency neurotomy for chronic cervical zygapophyseal-joint pain. N Engl J Med.

[15] McCormick Z.L., Cohen S.P., Walega D.R., Kohan L. (2021). Technical considerations for genicular nerve radiofrequency ablation: optimizing outcomes. Reg Anesth Pain Med.

[16] Tate Q., Meiling J.B., Burnham T.R., Conger A., McCormick Z.L. (2022 Mar 2). A pilot study of an expanded genicular nerve radiofrequency ablation protocol for the treatment of chronic knee pain. Pain Med.

[17] Koshi E., Cheney C.W., Sperry B.P., Conger A., McCormick Z.L. (2020 Dec 25). Genicular nerve radiofrequency ablation for chronic knee pain using a three-tined electrode: A technical description and case seriesn. Pain Med.

[18] Chen Y., Vu T.H., Chinchilli V.M., Farrag M., Roybal A.R., Huh A., Cohen Z.O., Becker A.B., Arvanaghi B., Agrawal M., Ogden J., Cohen S.P. (2021 Apr). Clinical and technical factors associated with knee radiofrequency ablation outcomes: a multicenter analysis. Reg Anesth Pain Med.

[19] Koshi E., Meiling J.B., Conger A.M., McCormick Z.L., Burnham T.R. (2022). Long-term clinical outcomes of genicular nerve radiofrequency ablation for chronic knee pain using a three-tined electrode for expanded nerve capture. Interventional Pain Medicine.

[20] Caragea M., Woodworth T., Curtis T., Blatt M., Cheney C., Brown T., Carson D., Kuo K.T., Randall D., Huang E.Y., Carefoot A., Teramoto M., Mills M., Cooper A., Burnham T., Conger A., McCormick Z.L. (2023 Dec 1). enicular nerve radiofrequency ablation for the treatment of chronic knee joint pain: a real-world cohort study with evaluation of prognostic factors. Pain Med.

[21] A Sequenced Strategy for Improving Outcomes in People With Knee Osteoarthritis Pain. Available at: https://clinicaltrials.gov/study/NCT04504812.

[22] Cotten A., Dewatre F., Cortet B., Assaker R., Leblond D., Duquesnoy B., Chastanet P., Clarisse J. (1996). Percutaneous vertebroplasty for osteolytic metastases and myeloma: effects of the percentage of lesion filling and the leakage of methyl methacrylate at clinical follow-up. Radiology.

[23] Leclair A., Gangi A., Lacaze F., Javier R.M., Bonidan O., Kempf J.F., Bonnomet F., Limbach F.X., Kuntz J.L., Dietmann J.L., Sibilia J. (2000). Rapid chondrolysis after an intra-articular leak of bone cement in treatment of a benign acetabular subchondral cyst: an unusual complication of percutaneous injection of acrylic cement. Skeletal Radiol.

[24] Fogel G., Musie J., Phillips T.R., Shonnard M., Youssef S., Hirsch J.A., Beall D.P. (2023 Sep 26). Assessment and management of patients developing low energy vertebral compression fractures following basivertebral nerve ablation. Pain Med.

[25] Shaikh W., Miller S., McCormick Z.L., Patel P.M., Teramoto M., Walega D.R. (2023). Chemical neurolysis of the genicular nerves for chronic refractory knee pain: an observational cohort study. Pain Med.

[26] Risso R.C., Miller S., McCormick Z.L., Patel P.M., Teramoto M., Walega D.R. (2023). Chemical neurolysis of the genicular nerves for chronic refractory knee pain: an observational cohort study. Pain Med.

[27] Yildiz G., Perdecioglu G.R.G., Yuruk D., Can E., Akkaya O.T. (2023). Comparison of the efficacy of genicular nerve phenol neurolysis and radiofrequency ablation for pain management in patients with knee osteoarthritis. Korean J Pain.

[28] Shealy C.N. (1975). Percutaneous radiofrequency denervation of spinal facets: treatment for chronic back pain and sciatica. J Neurosurg.

